# Draft genome sequences of two oriental melons, *Cucumis melo* L. var. *makuwa*

**DOI:** 10.1038/s41597-019-0244-x

**Published:** 2019-10-22

**Authors:** Ah-Young Shin, Namjin Koo, Seungill Kim, Young Mi Sim, Doil Choi, Yong-Min Kim, Suk-Yoon Kwon

**Affiliations:** 1 Plant Systems Engineering Research Center, Korea Research Institute of Bioscience and Biotechnology (KRIBB), Daejeon, 34141 Korea; 2 Biosystems and Bioengineering Program, University of Science and Technology, Daejeon, 34113 Korea; 3 Korean Bioinformation Center (KOBIC), Korea Research Institute of Bioscience and Biotechnology (KRIBB), Daejeon, 34141 Korea; 4 Department of Plant Science, College of Agriculture and Life Sciences, Seoul National University, Seoul, 08826 Korea; 5 Department of Plant Science and Plant Immunity Research Center, College of Agriculture and Life Sciences, Seoul National University, Seoul, 08826 Korea

**Keywords:** Plant breeding, Plant genetics

## Abstract

Oriental melon (*Cucumis melo* L. var. *makuwa*) is one of the most important cultivated cucurbits, and is grown widely in Northeast Asian countries. With increasing interest in its biological properties and economic importance, oriental melon has become an attractive model crop for studying various horticultural traits. A previous genome sequence of the melon was constructed from a homozygous double-haploid line. Thus, individual reference genomes are required to perform functional studies and further breeding applications. Here, we report draft genome sequences of two oriental melons, Chang Bougi and SW3. The assembled 344 Mb genome of Chang Bougi was obtained with scaffold N50 1.0 Mb, and 36,235 genes were annotated. The 354 Mb genome of SW3 was assembled with scaffold N50 1.6 Mb, and has 38,173 genes. These newly constructed genomes will enable studies of fruit development, disease resistance, and breeding applications in the oriental melon.

## Abstract (summary)

**Table:** 

Measurement(s)	DNA
Technology Type(s)	DNA sequencing
Factor Type(s)	reference genome
Sample Characteristic - Organism	Cucumis melo

Machine-accessible metadata file describing the reported data: 10.6084/m9.figshare.9939629

## Background & Summary

The oriental melon (*Cucumis melo* L. var. *makuwa*), one of the most important annual diploid crops within the Cucurbitaceae family, is grown largely in Northeast Asian countries, including Korea, China, and Japan. It is cultivated primarily for its fruit, which generally has a sweet aromatic flavor and contains soluble sugars, organic acids, minerals, and vitamins^1–3^. Traits of the fruit, such as shape, skin color, flesh color, and sugar content, are highly variable. Because its economic importance and interest in its biological properties have increased, oriental melon has become an attractive model crop for the study of various traits.

Reference genomes from genetically diverse individuals provide insights into genome structures, genome evolution, and diversification within the genus and species. For instance, precise comparison of genome structures and analyses about lineage-specific evolution of gene families in the genus *Capsicum* became possible through the completion of multiple reference genomes^4^. In the case of melon, a previous reference genome was constructed from the homozygous DHL92 double-haploid line^5^, and subsequent improvements to the genome assembly and annotations were reported^6^. To carry out functional studies, evolutionary studies of gene families, link genetic markers to desirable traits, and further breeding applications in the oriental melon, multiple reference genomes will be required.

Here, we report the construction of draft genomes of two oriental melon types, Chang Bougi and SW3. Chang Bougi, a Korean landrace, is a new source for the breeding of resistance to *Cucumber Green Mottle Mosaic Virus* (CGMMV), which causes mosaicism in leaves and deterioration of fruits, leading to severe yield and quality losses of cucurbit crops worldwide^7^. The high-quality breeding line SW3, from NongWoo Bio Company, contains deep-yellow and oval-type fruits with high sugar content.

**Fig. 1:**
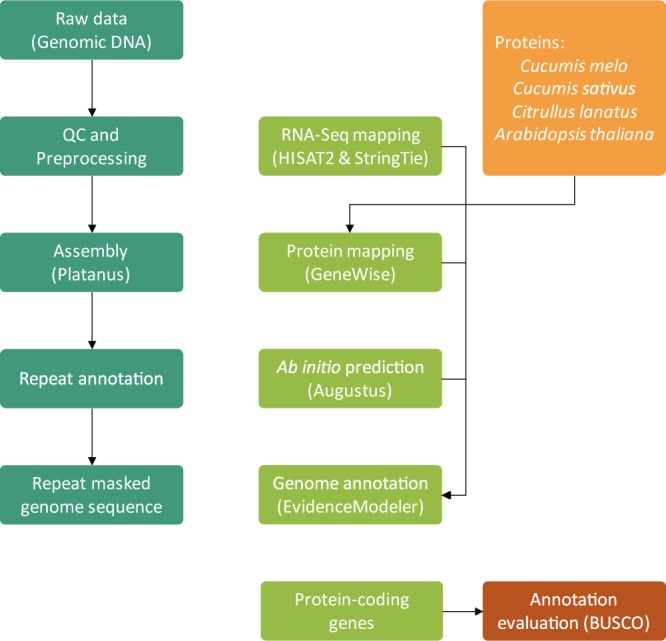
Overview of the pipeline of the study.

**Fig. 2:**
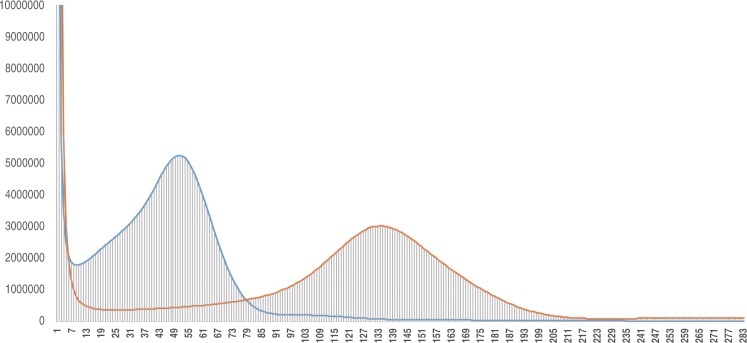
Distribution of 19-mers in raw sequence data from two oriental melon genomes. Distribution of 19-mers for Chang Bougi (blue) and SW3 (orange) are depicted. The *x-* and *y-*axes indicate frequency and volume of 19mers, respectively.

**Fig. 3:**
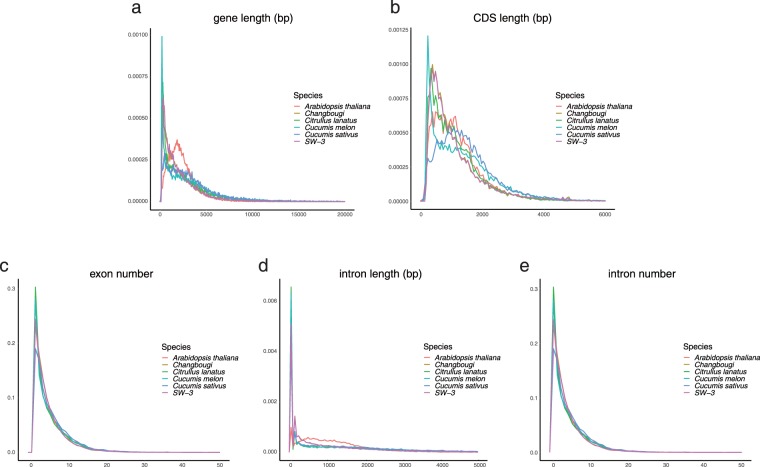
Comparisons of gene models for two oriental melon genomes and other genomes. (**a**) Gene length distribution (**b**) CDS length distribution (**c**) Exon number distribution (**d**) Intron length distribution (**e**) Intron number distribution. *x*-axis stands for length (bp) of gene (**a**), CDS (**b**) and intron (**d**) or numbers of exon (**c**) and intron (**e**), respectively. *y*-axis stands for ratio of genes.

**Fig. 4:**
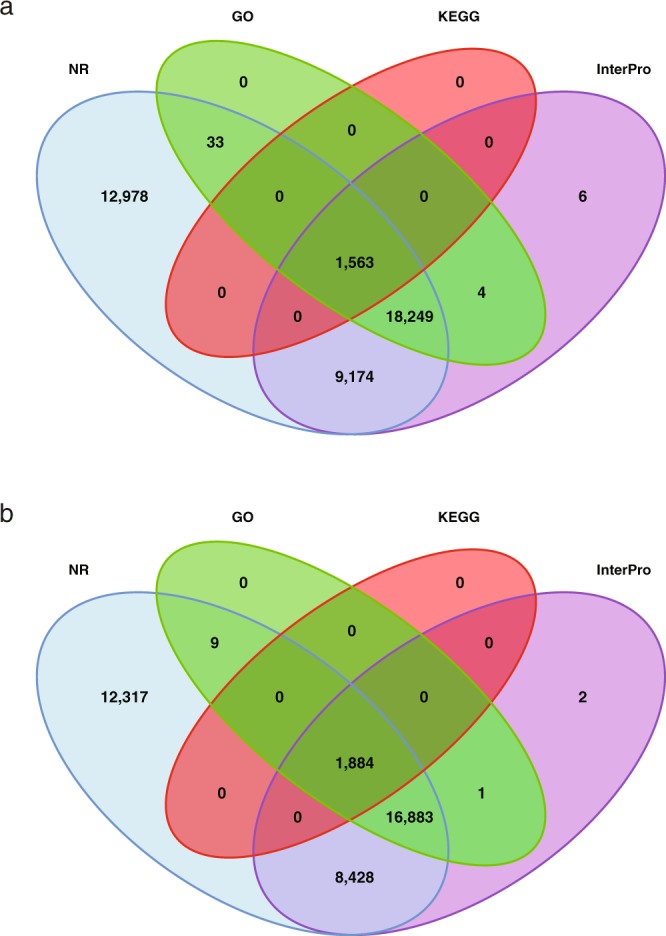
Venn diagram of the number of genes having functional annotation in Chang Bougi and SW3 genomes using multiple public databases. Functional annotation of Chang Bougi (**a**) and SW3 (**b**) were primarily performed using Blast2Go. For genes that remained unassigned by Blast2Go, we used NR, GO, KEGG, and InterPro to assign gene function.

Figure 1 presents an overview of the study. A combination of paired-end (PE) and mate-pair (MP) libraries were sequenced to generate 231× and 345× of genomic sequencing data^8^, respectively, for Chang Bougi and SW3 (Table 1). Genome assembly and annotation were then performed (Fig. 1). The assembled genome of Chang Bougi^9^ comprised 11,309 scaffolds totaling 344 Mb in length, with scaffold N50 of 1.0 Mb. For SW3, 7,202 scaffolds totaling 354 Mb in length were assembled^10^, with scaffold N50 of 1.6 Mb (Table 2). Repeat annotation was then carried out (Table 3). *K-*mer frequencies were calculated to provide information related to low frequencies, sequencing depth, level of heterozygosity, and genome size (Fig. 2)^11^. The estimated genome sizes of Chang Bougi and SW3 were 355 Mb and 373 Mb, respectively, which were similar to previously reported genome sizes^5^. A total of 36,235 and 38,173 genes were determined as final genes in Chang Bougi and SW3, respectively (Table 2 and Fig. 3). Then functional annotation of final gene models were performed (Table 4 and Fig. 4). Finally, we provide new reference genome of oriental melons for further analysis and breeding program.

**Table 1:** Metrics of raw Illumina datasets.

Library Type	Samples	Insert Size (bp)	Read Length (bp)	Coverage (×)
Paired-end	Chang Bougi	400	150	57.5
		800	150	43.7
Mate-pair		2,000	150	54.4
		5,000	150	34.6
		10,000	150	41.7
Paired-end	SW3	400	150	166.7
		800	150	50.3
Mate-pair		2,000	150	55.2
		5,000	150	39.8
		10,000	150	33.5

**Table 2:** Statistics of genome assembly and annotation.

	Chang Bougi	SW3
Number of scaffolds	11,309	7,202
Total length of scaffolds (Mbp)	344	354
N50 of scaffolds (Mbp)	1.0	1.6
Longest scaffold length (Mbp)	6.8	5.6
Number of contigs	43,251	29,154
Total length of contigs (Mbp)	325	346
N50 of contigs (kbp)	15	25
Longest contig length (kbp)	160	214
Number of genes	36,235	38,173
Average/total CDS lengths	1,083/39,426,107	1,107/42,780,742
Average exon/intron lengths	243/346	248/356

**Table 3:** Statistics of repeat annotation.

Type	Chang Bougi		SW3	
	Length (Mb)	Ratio (%)	Length	Ratio
DNA elements	39	11	37	10
LINE elements	4	1	5	1
SINE elements	0	0	0	0
LTR/Gypsy	29	8	36	10
LTR/Copia	31	9	34	10
LTR/Caulimoviridae	4	1	5	1
rDNA	0	0	0	0
Simple repeat	6	2	6	2
Others	2	1	3	1
Unclassified	64	19	68	19
Total	179	52	194	54

**Table 4:** Functional annotation of genes.

Database	Chang Bougi		SW3	
	Annotated Number	Annotated Percent (%)	Annotated Number	Annotated Percent (%)
NR	39,521	98.86	41,997	98.74
InterPro	27,198	68.03	28,996	68.17
GO	18,777	46.97	19,849	46.67
KEGG	1,884	4.71	1,563	3.67
Annotated	39,524	98.87	42,007	98.76
Total	39,977	—	42,535	—

## Methods

### DNA extraction and sequencing

Leaves of two oriental melons were harvested and frozen immediately in liquid nitrogen. Genomic DNA was extracted, and paired-end and mate-pair libraries for next-generation sequencing were constructed according to the manufacturer’s instructions (Illumina, San Diego, CA, USA). The quality of each library was validated using the KAPA SYBR FAST Universal 2× qPCR Master Mix (Kapa Biosystems, Boston, MA, USA). Each library was sequenced with the Illumina HiSeq 2500 platform.

### Genome assembly

Pre-processing analyses of raw sequences, using in-house pipeline and genome assembly, were performed as described in previous studies^4,12^. After pre-processing to remove erroneous sequences in raw data, remaining sequences in paired-end libraries were assembled using Platanus^13^, with parameters for Chang Bougi (-k 63 -c 5 -d 0.3 -t 40 -m 220) and for SW3 (-k 91 -c 5 -d 0.3 -t 44 -m 200). The scaffolding process was performed with Platanus, using paired-end and mate-pair sequences, with parameters for Chang Bougi (-l 3 -s 61 -u 0.2 -t 40), and for SW3 (-l 3 -u 0.2 -t 15). Remaining gaps were filled with Platanus and GapCloser version 1.10 (http://soap.genomics.org.cn/down/GapCloser_release_2011.tar.gz), using reads from the paired-end and mate-pair libraries. Finally, 344 Mb of Chang Bougi genomic sequence (96.9% of 355 Mb) and 354 Mb of SW3 genomic sequence (94.9% of 373 Mb) were assembled (Table 2).

### Repeat annotation

After construction of repeat libraries using the assembled Chang Bougi and SW3 genomes, repeat annotation was implemented using RepeatModeler and RepeatMasker (http://www.repeatmasker.org). A total of 179 Mb (52% of 355 Mb) and 194 Mb (54% of 373 Mb) of repeat sequences were detected in Chang Bougi and SW3, respectively (Table 3).

### Genome annotation

Annotation of the two genomes were performed using the KOBIC annotation pipeline (a modified PGA pipeline^14^), consisting of repeat masking, mapping of different protein sequence sets, and *ab initio* prediction performed by AUGUSTUS v3.2.2^15^. Transcript assembly was performed with the assembled genome by a reference-based algorithm using HISAT2^16^ and StringTie^17^. To generate protein-based gene models for consensus modeling, the protein sequences of *Arabidopsis thaliana* (TAIR10, http://www.arabidopsis.org), *Citrullus lanatus*^18^, *Cucumis melo*^5^, and *Cucumis sativus*^19^ were mapped using GeneWise v2.1^20^. AUGUSTUS was used for gene prediction in the two oriental melon genomes. To validate the predicted gene models, protein sequences from the genomes of *C*. *lanatus*, *C*. *melo*, *C*. *sativus*, and *A*. *thaliana* were used as queries in BLASTp, and erratic gene models were filtered with a BLASTp cut-off of query coverage ≥0.3. Also, the assembled transcripts were validated against the same four sets of protein sequences using tLBASTn, and filtered with cut-off values of query coverage ≥0.5 and subject coverage ≥0.3. The GeneWise gene models that remained were reformatted from GeneWise format to GFF3 data, and used to determine the consensus gene model via EVM^21^, which combines *ab initio* gene predictions with protein alignments into weighted-consensus gene structures (*ab initio* predictions = 1, protein alignment = 5, transcript alignment assemblies = 7). Ultimately, the final gene models included a total of 36,235 consensus genes for Chang Bougi and 38,173 consensus genes for SW3 (Table 2 and Fig. 3).

Further functional annotations were performed using the program Blast2Go^22^, including InterPro^23^, NR from NCBI, Kyoto Encyclopedia of Genes and Genomes (KEGG)^24^. Functional annotation of the final gene models (Table 4 and Fig. 4) predicted 2,093, 3,703, and 493 genes as hypothetical protein, uncharacterized protein, and unknown function, respectively, in the Chang Bougi genome. In the SW3 genome, respectively 2,245, 3,827, and 570 genes were predicted as hypothetical protein, uncharacterized protein, and unknown function.

## Data Records

All of the raw sequence reads produced by Illumina HiSeq 2500 have been deposited at NCBI Sequence Read Archive (SRA) under BioProject number PRJNA531526 (accession SRP191487)^8^ and BioSample from SAMN11368505 to SAMN11368524 (SAMN11368505 ~ SAMN11368515 for Chang Bougi; SAMN11368516 ~ SAMN11368524 for SW3). The Whole Genome Shotgun project of Chang Bougi have been deposited at DDBJ/ENA/GenBank under the accession number SSTD00000000^9^ under PRJNA531576 and SAMN11370205. The Whole Genome Shotgun project of SW3 have been deposited at DDBJ/ENA/GenBank under the accession number SSTE00000000^10^ under BioProject number PRJNA531478 and BioSample SAMN11381272.

## Technical Validation

### Detection and filtration of misannotated genes

EvidenceModeler predicted 39,977 and 42,535 consensus genes for Chang Bougi and SW3, respectively. We investigated these to detect misannotated genes, as recommended by NCBI GenBank, including genes containing internal stop codons, genes lacking a stop codon, frame-shifted genes, or erroneous start codons. A total of 3,742 and 4,362 misannotated genes were detected and masked in Chang Bougi and in SW3, respectively. Thus, 36,235 genes remained in the Chang Bougi genome, and 38,173 genes remained in SW3.

### Evaluation of genome annotation using BUSCO

BUSCO v3.0.2^25^ provides an assessment of assembled genome completeness based on the orthologous group, with single-copy genes from OrthoDB (http://www.orthodb.org), and using a hidden Markov model to profile amino acid alignments. For genome annotation assessments, we used 1,440 gene sets of orthologs conserved in embryophyta (Table 5). The results showed that nearly all of these core genes/orthologs were present in the genomes of Chang Bougi (85.28%) and SW3 (86.81%).

**Table 5:** The presence and completeness of universally conserved single-copy genes in Chang Bougi and SW3 (BUSCO) genomes.

	Chang Bougi	SW3
Complete BUSCOs (C)	1228	1250
Complete and single-copy BUSCOs (S)	1199	1220
Complete and duplicated BUSCOs (D)	29	30
Fragmented BUSCOs (F)	103	87
Missing BUSCOs (M)	109	103

### Comparison of gene sets in the genomes of oriental melons Chang Bougi and SW3 with those in the genomes of melon (DHL92 v3.6.1) and cucumber

**Fig. 5:**
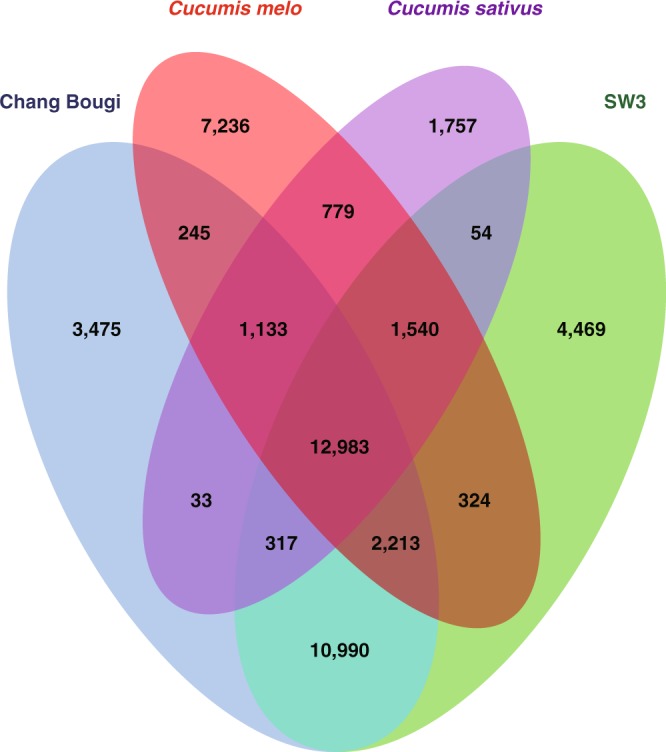
Distribution of orthologous gene families of *Cucumis melo* (DHL92 v3.6.1), *Cucumis sativus*, Chang Bougi, and SW3 genomes. A total of 113,006 sequences were clustered into 30,738 groups. Each panel shows the number of clustered genes for that genome.

To compare gene sets between oriental melons and previously reported cucurbit genomes, orthologous and paralogous genes were detected in melon genome (DHL 92 v3.6.1), Chang Bougi, SW3, and cucumber (*C*. *sativus*) using the program OrthoFinder^26^. A total of 113,006 sequences were clustered into 30,738 groups, with 3,475 and 4,469 singleton genes detected in Chang Bougi and in SW3, respectively (Fig. 5). Fewer singleton genes might be expected in the two oriental melons than in the melon genome, which was constructed from a homozygous DHL92 double-haploid line, derived from a cross between Korean landraces of oriental melon (Songwhan Chamoe, PI 161375) and melon (Piel de Sapo). In addition, 2,213 genes were determined as common among melon and the two oriental melons, and 12,983 genes were detected in all four genomes. Functional investigation of singleton genes of Chang Bougi and SW3 indicated that 869 and 1,112 of genes were functionally unknown genes, respectively.

## Data Availability

The sequence data were generated using software provided by the sequencing platform manufacturer, and were processed with publicly available software and recommended settings, as cited in this report. No custom computer codes were generated in this work.
